# Ultrasound-guided percutaneous liver biopsy: A review of what operators need to know

**DOI:** 10.1097/MD.0000000000038673

**Published:** 2024-07-26

**Authors:** Husain Alturkistani, Abdullah H. Alsergani, Meshari Alzeer, Anas Alturkistani, Renad Zaini, Salem Bauones

**Affiliations:** a Department of Interventional Radiology, College of Medicine, King Saud University, Riyadh, Saudi Arabia; b Department of Radiology, King Faisal Specialist Hospital and Research Center, Riyadh, Saudi Arabia; c College of Medicine, King Saud University, Riyadh, Saudi Arabia; d College of Medicine, University of Jeddah, Riyadh, Saudi Arabia; e College of Medicine, Princess Nourah University, Riyadh, Saudi Arabia; f Department of Interventional Radiology, King Fahad Medical City, Riyadh, Saudi Arabia.

**Keywords:** interventional radiology, liver biopsy, liver cirrhosis, radiology, UG-PLB, ultrasound guided, ultrasound-guided percutaneous liver biopsy

## Abstract

Ultrasound-guided percutaneous liver biopsy (UG-PLB) is a commonly performed procedure for the diagnosis and monitoring of various liver diseases. The objective of this article is to present the most important information relating to UG-PLB from the perspective of interventional radiologists in a clear and concise fashion, referencing the most influential papers in current literature. This paper gives a brief overview of the history of the procedure and its conception, as well as the most common indications, contraindications, complications, and a technical overview of the most common techniques and equipment that are used by UG-PLB operators.

## 1. Introduction

Liver biopsy is the standardized procedure for obtaining hepatic tissue samples for the sake of histopathological examination. It is considered the gold standard investigation of many diagnostic modalities in terms of investigating various liver pathologies.^[1]^ The significance of liver biopsy lies in its specificity and sensitivity, which was found to be 89% and 93% for most disease use cases.^[2]^ It is considered the most specific investigation for monitoring the nature and severity of liver diseases, as well as disease progression and response to various treatments.^[3]^ The first ever Liver biopsy has been credited to Paul Ehrlich, who performed the procedure in 1883.^[4]^ Although the first published report of the procedure was written in 1923,^[5]^ the procedure was popularized and standardized after publication by Menghini^[6]^ “One-second needle biopsy of the liver” in 1958. Technological breakthroughs throughout the years have allowed the procedure to be considered fairly routine due to the implementation of image guidance. This can be achieved through ultrasound (US), computed tomography guidance, or through magnetic resonance imaging.^[6]^ Accumulated experience and published literature both agree that image guidance, mainly under US, is far superior to the blind approach, as it has shown to lower complication rates and increase success rates of the diagnostic yields of the procedure. Therefore, it has been considered very difficult to justify the incidence of complications during a blind liver biopsy that could have been avoided when it can be avoided through image guidance.^[1]^ Liver biopsies can be done through different approaches, the most common of which are the percutaneous, endoscopic, and transjugular methods. The percutaneous approach is considered the most conventional and widely used method, while the endoscopic and transjugular approaches are considered newer emerging approaches, all of which have their respective indications, advantages, and disadvantages.^[7]^ Although the procedure can be done under computed tomography guidance, US is usually preferred because it provides the only instantaneous visualization of needle position without exposing the patient to any ionizing radiation.^[8]^ US-guided percutaneous liver biopsies (UG-PLBs) are most commonly performed by gastroenterologists and interventional radiologist, both of which have similar success and complication rates,^[9]^ although the procedure has been increasingly performed by interventional radiologists rather than gastroenterologists in recent years.^[9]^ The objective of this paper is to provide a descriptive review of UG-PLB and to provide a technical overview from the perspective of interventional radiologists.

## 2. Indications

UG-PLB is most commonly performed for the diagnosis and monitoring of chronic hepatitis, as it is still considered the “gold standard” investigation.^[10]^ It has also been suggested that since the most common cause of chronic hepatic disease is chronic viral infection through hepatitis B and C, the most likely cause the procedure will be done on patients would therefore be chronic viral hepatitis.^[11,12]^ Other common indications of liver biopsy include the diagnosis of hepatic steatosis, infectious and infiltrative liver disease, Wilson disease, hepatic storage diseases, liver manifestations of amyloidosis in select cases, and for the diagnosis of autoimmune liver pathologies such as primary biliary cholangitis, primary sclerosing cholangitis, and Autoimmune hepatitis.^[13]^ Liver biopsy is also performed for the investigation of persistently elevated liver function tests without a clear etiology, and as part of the workup of fever of unknown origin.^[14]^

## 3. Contraindication

### 3.1. Absolute contraindications

The situations where UG-PLB should not be performed are well described in the literature.^[15]^ Logistical contraindications include the inability of undergoing informed consent, the lack of full cooperation with healthcare staff, and refusal of patients to accept blood transfusions in case it is required during the procedure. Medical contraindications include significant coagulopathy or other bleeding disorders that cannot be corrected prior to the procedure, recent nonsteroidal anti-inflammatory drug use, the presence of vascular liver tumors, the presence of hydatid cyst, and severe ascites.^[3,15]^

### 3.2. Relative contraindications

The relative contraindications that should be considered at a case-by-case basis include the following: morbid obesity causing functional limitation to the operator, mild-to-moderate ascites, infection under the level of the diaphragm taking into consideration the severity of infection and the reason of the liver biopsy, and amyloidosis.^[15,16]^

## 4. Needles

The type of needles used for liver biopsies can be classified into 2 main subtypes: cutting needles and aspiration needles. There are also spring-loaded trigger devices that can be used in conjunction with the aforementioned subtypes to ensure easier sample acquisition called “guns.” Cutting needles are needles that retrieve a sliced segment from the biopsied tissue, whereas aspiration needles rely on suctioning the biopsied tissue to retrieve a histopathological sample. Many studies have tried investigating the differences between the efficacies of the 2 types of needles. The literature has been consistently mixed on this specific topic. Some studies reported no significant differences in complication and success rates.^[17,18]^ Others reported superior sample acquisition with cutting needles in comparison to aspiration needles.^[19]^ A group of studies found specific complications to be associated with each needle type, where cutting needles were associated with hemorrhagic complications, biliary peritonitis, and pneumothorax, and aspiration needles were more frequently associated with visceral perforation and sepsis. All in all, the agreed upon consensus of the choice of needle depends on the preference of the proceduralist, the availability of equipment, and the clinical context, where cutting needles are to be used in cases of cirrhosis for example.^[16]^ Recently, the spring-loaded cutting biopsy needle has become widely favored by operators over other needle combinations, mainly because it is quicker and therefore allows less time for respiratory motion to be a significant hindrance to sample acquisition.^[3]^

## 5. Approach

There exist 2 possible approaches for UG-PLB. The transthoracic and subcostal routes. The transthoracic approach involved the introduction of the biopsy needle through the intercostal area, usually between the 7th and 8th ribs at the midaxillary line, whereas the subcostal approach is the introduction of the biopsy needle at the subcostal margin. The subcostal approach is not as commonly used because it necessitates that the liver extends past the costal margin.^[20]^ Today, most physicians opt for the transthoracic instead of the subcostal approach for all of their cases.

## 6. Patient preparation

### 6.1. Inpatient versus outpatient procedure

UG-PLB has initially been a purely inpatient procedure until the late '60s, where outpatient procedures were popularized in the United States.^[20]^ A satisfaction rate of 91% was achieved in postprocedural surveys, while admission rates due to complications were found to be 2% to 3%.^[21]^ The latest consensus of the American Gastroenterological Association regarding the choice of inpatient versus outpatient UG-PLB is that patients undergoing outpatient UG-PLB should have no comorbidities that increase the risk of complications such as biliary obstruction, heart failure, older age, and the presence of malignancy. It is also recommended that outpatient cases be done in close proximity to inpatient facilities should the need arise.^[16]^ Advancements over the past decades have allowed liver biopsies to be typically done on an outpatient or “same day” basis.^[3]^

### 6.2. Pre-op preparation

Prior to the procedure, a detailed written consent form detailing the risks and benefits of the procedure and including indications and therapeutic consequences should be signed by the patient. Common pre-op investigations include complete blood count, a full coagulation profile including prothrombin time and/or international normalized ratio (INR), activated partial thromboplastin, and bleeding time. Some experts also recommend having the patient blood typed in case the need arises for blood transfusions.^[22]^ A minimum platelet count of 50,000 and an INR of <1.6 is usually required before the procedure. In case these cutoffs are not met, the usage of vitamin K, fresh frozen plasma, cryoprecipitate, and platelet transfusion should be considered by operators depending on the overall clinical picture and how urgently the biopsy is needed. Patients should be told to discontinue their antiplatelet medication a minimum of 7 days prior to the procedure, while heparin and warfarin should be stopped a minimum of 6 hours and 5 days before the procedure, respectively.^[23]^

## 7. Technique

**Figure 1.**
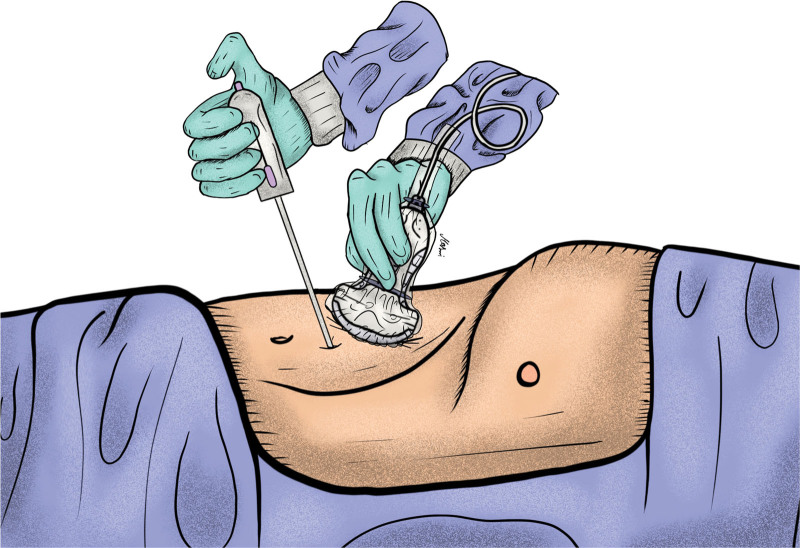
Illustration of ultrasound-guided percutaneous liver biopsy.

**Figure 2.**
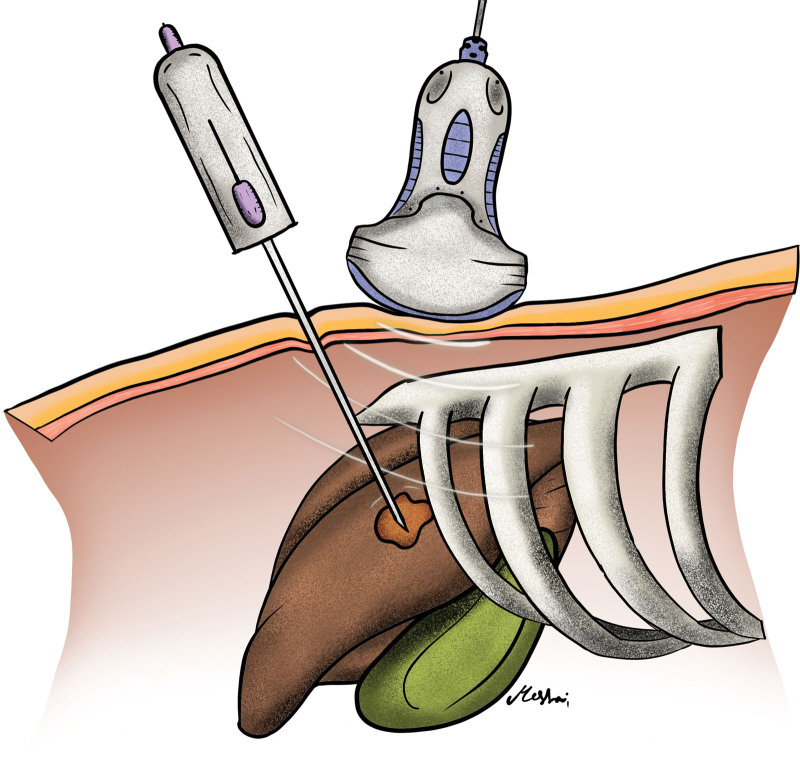
Illustration of adequate probe and needle positioning.

**Figure 3.**
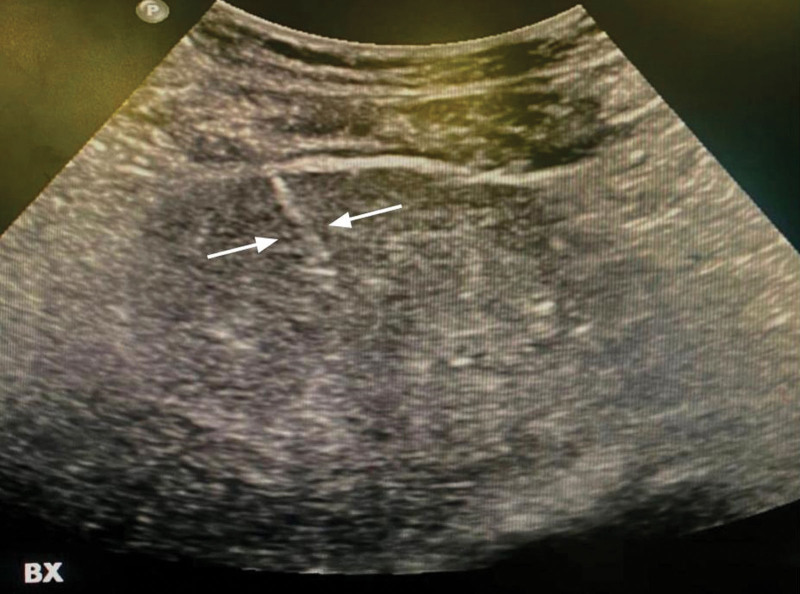
Frozen ultrasound image of the needle (white arrow) in the liver.

The technique of UG-PLB from the perspective of interventional radiologists using a spring-loaded cutting biopsy includes the following. Upon the patient’s arrival to the Interventional Radiology suite, double checking that the procedure indication, duration, technique, and complications were previously explained to the patient thoroughly. Preliminary US scans should be done prior to the first puncture to plan the site of needle insertion and to determine the direction of the biopsy needle while advancing it. Although placing the patients in sufficient in most use cases, patients may be placed in the oblique, decubitus, and even prone position depending on the exact biopsy target site. The right upper abdominal quadrant is then completely sterilized, and A sterile perforated sheet is applied over the area. A 5- to 7-MHz curved US probe is then prepared by covering it with a sterile probe cover and applying US gel. The operator should also be completely sterile through the usage of sterile gowns and gloves. Figure 1 illustrates an adequate sterile field and correct placement of the US probe and needle. The procedure starts with the repetition of the preliminary US scans done at the beginning. Local anesthesia is then given under US guidance up to the capsule of liver preferably with a 23-gauge needle. A 16- or 18-gauge disposable automatic spring-loaded true cut core biopsy needle is then advanced attentively toward the xiphoid process under US guidance in the superior portion of the intercostal region between the 7th and 8th ribs along the midaxillary line as to avoid neurovascular structures that cross along the lower border of each rib. Figure 2 illustrates the tip of the biopsy needle in the liver parenchyma. The tip of the biopsy needle should be visualized at all times while advancing it, to avoid blood vessels and biliary ducts. Complete liver parenchyma should be seen at least 2 to 3 cm after advancing the tip of the needle as seen in Figure 3. The biopsy needle is then fired to get the sample in the pathology containers. At least 2 full-core biopsies should be obtained, spanning a minimum of 20 mm longitudinally and 1 mm vertically. A final US scan is done afterward to check for any immediate complications. Post-op care includes strict bed rest for at least 4 hours, with vital signs checkup every 30 minutes for the first 2 hours then every hour for the next 2 hours. The site of the biopsy should also be monitored for any signs of hematoma or bleeding.^[24]^

## 8. Complication

UG-PLB is considered a very safe procedure according to its low complication rate. It has been reported that the complication rates of the procedure are between 0.3% and 0.5% in most patients. The most common of which is bleeding.^[25,26]^

### 8.1. Major complication

Major complications are complications that require major medical interventions and are associated with significant morbidity and mortality to patients. Bleeding is by far the most frequent serious adverse effect of UG-PLB. Hepatic puncture causes bleeding, which manifests as hemoperitoneum, subcapsular hematoma, or hemobilia. Bleeding due to UG-PLB rarely requires anything more than supportive treatment and observation, although transfusion and surgical intervention may be indicated in decompensating patients. Thus, the majority of bleeding following biopsy is mild and only needs to be watched closely.^[25]^ The most significant risk factors for bleeding have been shown to be older age, the presence of malignancy, increased number of biopsy passes, and coagulation disorders.^[17,27]^ In such patients, employing plugged biopsies may be beneficial in lowering the inflated bleeding rates. Plugged biopsies involve injecting a gelfoam slurry into the biopsy puncture using a coaxial needle, which acts as a temporary embolic agent. The procedure is then followed by tight compression. Plugged liver biopsies have been shown to significantly reduce the risk of bleeding associated with liver biopsies.^[28]^

The next most frequent major complications requiring hospital admission are severe intractable pain, followed by pneumothorax, and gallbladder puncture. It has also been shown that 60% of the complications happen within the first 2 hours, 82% within the first 10 hours, and 96% within the first 24 hours of the procedure.^[26]^

### 8.2. Minor complications

Minor complications are defined as those that require little-to-no medical intervention and do not result in major morbidity and mortality. The vast majority of reported complications of UG-PLB are minor ones. Minor complications occur in 3% to 5% of UG-PLB. Postbiopsy pain at the site of the procedure or referred pain to the left shoulder is the most commonly reported complication of UG-PLB overall. Risk factors influencing the occurrence of pain include premedication with midazolam and fentanyl and high operator experience level. It is thought that the reason UG-PLB by more experienced operators is reported at lower rates is because of the accurate placement of anesthesia in the liver capsule. Further emphasizing the need for highly experienced and well-trained operators.^[29]^ Another common minor complication is the incidence of a vasovagal reaction. This complication has been reported to occur in 1% to 3% of UG-PLB. The only intervention that was found to cause a significant reduction in its incidence is premedication with midazolam.^[30]^

### 8.3. Risk factors

To aid in the transition from being a purely inpatient procedure to being a procedure that was a routine day case, many studies were done to assess any variables or factors that may increase the risk of complications and adverse events in order to evaluate the possibility of controlling for them and consequently lowering the complication rates. As such, patients who had more advanced liver disease characterized by lower platelet counts and higher INR and patients with endoscopically proven esophageal varices were most likely to suffer from complications after the procedure.^[25]^ In terms of factors unrelated to patients, operator experience, larger needles, and increased number of biopsy passes were also found to be predictors of the incidence of complications. Interestingly, the biopsy approach (transthoracic vs subcostal) and the needle subtype were not consistently found to be a significant risk factor for increasing the rate of complications.^[29]^

## 9. Summary/conclusion

UG-PLB is an important procedure that provides invaluable input regarding disease diagnosis and disease status. UG-PLB is a relatively simple procedure that has been shown to be very safe with minimal complications. The usage of US guidance should be a staple when conducting the procedure, as it further lowers the complication rate and ensures the success of the procedure to a large extent.

## Author contributions

**Conceptualization:** Husain Alturkistani, Abdullah H. Alsergani, Meshari Abdulaziz Alzeer, Anas Alturkistani, Renad Hatim Zaini, Salem Bauones.

**Investigation:** Husain Alturkistani, Abdullah H. Alsergani, Meshari Abdulaziz Alzeer, Renad Hatim Zaini, Salem Bauones.

**Methodology:** Husain Alturkistani, Abdullah H. Alsergani, Meshari Abdulaziz Alzeer, Renad Hatim Zaini, Salem Bauones.

**Project administration:** Husain Alturkistani, Abdullah H. Alsergani, Meshari Abdulaziz Alzeer, Salem Bauones.

**Supervision:** Husain Alturkistani, Abdullah H. Alsergani, Salem Bauones.

**Validation:** Husain Alturkistani, Abdullah H. Alsergani, Meshari Abdulaziz Alzeer, Anas Alturkistani, Salem Bauones.

**Visualization:** Husain Alturkistani, Abdullah H. Alsergani, Meshari Abdulaziz Alzeer, Anas Alturkistani.

**Writing—original draft:** Husain Alturkistani, Abdullah H. Alsergani, Meshari Abdulaziz Alzeer, Anas Alturkistani, Renad Hatim Zaini, Salem Bauones.

**Writing—review & editing:** Husain Alturkistani, Abdullah H. Alsergani, Meshari Abdulaziz Alzeer, Anas Alturkistani, Renad Hatim Zaini, Salem Bauones.

**Data curation:** Abdullah H. Alsergani, Anas Alturkistani, Renad Hatim Zaini.

## References

[1] Al Knawy B Shiffman M. Percutaneous liver biopsy in clinical practice. Liver Int. 2007;27:1166–73. 17919227 10.1111/j.1478-3231.2007.01592.x

[2] Joy D Scott BB. To perform or not to perform liver biopsy: an alternative view. Gut. 2003;52:610. 10.1136/gut.52.4.610 PMC1773598 12631681

[3] Rockey DC Caldwell SH Goodman ZD Nelson RC Smith AD; American Association for the Study of Liver Diseases. American Association for the Study of Liver Diseases. Liver biopsy. Hepatology. 2009;49:1017–44. 19243014 10.1002/hep.22742

[4] Von Frerichs F. “Über den diabetes, Berlin.”. August Hirschwald. 1884;1:113.

[5] Bingel A. “Ueber die parenchympunktion der leber.”. Verh Dtsch Ges Inn Med.1923;35:210–2.

[6] Menghini G. One-second needle biopsy of the liver. Gastroenterology. 1958;35:190–9. 13562404

[7] McCarty TR Bazarbashi AN Njei B Ryou M Aslanian HR Muniraj T. Endoscopic ultrasound-guided, percutaneous, and transjugular liver biopsy: a comparative systematic review and meta-analysis. Clin Endosc. 2020;53:583–93. 33027584 10.5946/ce.2019.211 PMC7548145

[8] Copel L Sosna J Kruskal JB Kane RA. Ultrasound-guided percutaneous liver biopsy: indications, risks, and technique. Surg Technol Int. 2003;11:154–60. 12931297

[9] Potter C Hogan MJ Henry-Kendjorsky K Balint J Barnard JA. Safety of pediatric percutaneous liver biopsy performed by interventional radiologists. J Pediatr Gastroenterol Nutr. 2011;53:202–6. 21788763 10.1097/MPG.0b013e3182183012

[10] Sporea I Popescu A Sirli R. Why, who and how should perform liver biopsy in chronic liver diseases. World J Gastroenterol. 2008;14:3396–402. 18528937 10.3748/wjg.14.3396 PMC2716594

[11] Lai CL Ratziu V Yuen MF Poynard T. Viral hepatitis B. Lancet. 2003;362:2089–94. 14697813 10.1016/S0140-6736(03)15108-2

[12] Global surveillance and control of hepatitis C. Report of a WHO consultation organized in collaboration with the Viral Hepatitis Prevention Board, Antwerp, Belgium. J Viral Hepat. 1999;6:35–47. 10847128

[13] Vijayaraghavan GR David S Bermudez-Allende M Sarwat H. Imaging-guided parenchymal liver biopsy: how we do it. J Clin Imaging Sci. 2011;1:30. 21966627 10.4103/2156-7514.82082 PMC3177433

[14] Johnson KD Laoveeravat P Yee EU Perisetti A Thandassery RB Tharian B. Endoscopic ultrasound guided liver biopsy: recent evidence. World J Gastrointest Endosc. 2020;12:83–97. 32218888 10.4253/wjge.v12.i3.83 PMC7085945

[15] Chan M Navarro VJ. Percutaneous liver biopsy. April 10, 2023. In: StatPearls. Treasure Island, FL: StatPearls Publishing; 2023. 31985939

[16] Grant A Neuberger J. Guidelines on the use of liver biopsy in clinical practice. British Society of Gastroenterology. Gut. 1999;45(Suppl 4):IV1–IV11. 10485854 10.1136/gut.45.2008.iv1 PMC1766696

[17] Midia M Odedra D Shuster A Midia R Muir J. Predictors of bleeding complications following percutaneous image-guided liver biopsy: a scoping review. Diagn Interv Radiol. 2019;25:71–80. 30644369 10.5152/dir.2018.17525 PMC6339629

[18] Scheimann AO Barrios JM Al-Tawil YS Gray KM Gilger MA. Percutaneous liver biopsy in children: impact of ultrasonography and spring-loaded biopsy needles. J Pediatr Gastroenterol Nutr. 2000;31:536–9. 11144439 10.1097/00005176-200011000-00015

[19] Skamel HJ Hanusch A Mathias K. CT-gesteuerte Punktionen: Feinnadel-Aspirations-Biopsie versus automatisierte koaxiale Schneid-Biopsie [CT-guided punctures: fine-needle biopsy vs. automated co-axial cutting biopsy]. Aktuelle Radiol. 1998;8:273–7. 9894526

[20] Perrault J McGill DB Ott BJ Taylor WF. Liver biopsy: complications in 1000 inpatients and outpatients. Gastroenterology. 1978;74:103–6. 618417

[21] Douds AC Joseph AE Finlayson C Maxwell JD. Is day case liver biopsy underutilised? Gut. 1995;37:574–5. 7489948 10.1136/gut.37.4.574 PMC1382913

[22] Mueller M Kratzer W Oeztuerk S. Percutaneous ultrasonographically guided liver punctures: an analysis of 1961 patients over a period of ten years. BMC Gastroenterol. 2012;12:173. 23216751 10.1186/1471-230X-12-173 PMC3552862

[23] Atwell TD Smith RL Hesley GK. Incidence of bleeding after 15,181 percutaneous biopsies and the role of aspirin. AJR Am J Roentgenol. 2010;194:784–9. 20173160 10.2214/AJR.08.2122

[24] Winter TC Lee FT Jr. Hinshaw JL. Ultrasound-guided biopsies in the abdomen and pelvis. Ultrasound Q. 2008;24:45–68. 18362552 10.1097/RUQ.0b013e318168c869

[25] Seeff LB Everson GT Morgan TR. HALT–C Trial Group. Complication rate of percutaneous liver biopsies among persons with advanced chronic liver disease in the HALT-C trial. Clin Gastroenterol Hepatol. 2010;8:877–83. 20362695 10.1016/j.cgh.2010.03.025 PMC3771318

[26] Dotan Y Carlebach M Zuckerman E Maruf M Schiff E. Delayed bleeding after percutaneous liver biopsy. Eur J Case Rep Intern Med. 2016;3:000359. 30755857 10.12890/2016_000359 PMC6346949

[27] McGill DB Rakela J Zinsmeister AR Ott BJ. A 21-year experience with major hemorrhage after percutaneous liver biopsy. Gastroenterology. 1990;99:1396–400. 2101588 10.1016/0016-5085(90)91167-5

[28] Singhal S Pradeep MD Inuganti S Botcha S Deepashree DT Uthappa MC. Percutaneous ultrasound-guided plugged liver biopsy - a single-centre experience. Pol J Radiol. 2021;86:e239–45. 34093921 10.5114/pjr.2021.105852 PMC8147717

[29] Sparchez Z. Complications after percutaneous liver biopsy in diffuse hepatopathies. Rom J Gastroenterol. 2005;14:379–84. 16400355

[30] Sporea I Popescu A Stirli R Danila M Strain M. Ultrasound assisted liver biopsy for the staging of diffuse chronic hepatopathies. Rom J Gastroenterol. 2004;13:287–90. 15624025

